# Correlations between meteorological parameters and prostate cancer

**DOI:** 10.1186/1476-072X-9-19

**Published:** 2010-04-21

**Authors:** Sophie St-Hilaire, Sylvio Mannel, Amy Commendador, Rakesh Mandal, DeWayne Derryberry

**Affiliations:** 1 Department of Biological Sciences, Idaho State University, Pocatello, ID, 83209, USA; 2 Department of Geosciences, Idaho State University, Pocatello, ID 83209, USA; 3 Department of Health and Nutrition, Idaho State University, Pocatello, ID 83209, USA; 4 Mathematics Department, Idaho State University, Pocatello, ID 83209, USA

## Abstract

**Background:**

There exists a north-south pattern to the distribution of prostate cancer in the U.S., with the north having higher rates than the south. The current hypothesis for the spatial pattern of this disease is low vitamin D levels in individuals living at northerly latitudes; however, this explanation only partially explains the spatial distribution in the incidence of this cancer. Using a U.S. county-level ecological study design, we provide evidence that other meteorological parameters further explain the variation in prostate cancer across the U.S.

**Results:**

In general, the colder the temperature and the drier the climate in a county, the higher the incidence of prostate cancer, even after controlling for shortwave radiation, age, race, snowfall, premature mortality from heart disease, unemployment rate, and pesticide use. Further, in counties with high average annual snowfall (>75 cm/yr) the amount of land used to grow crops (a proxy for pesticide use) was positively correlated with the incidence of prostate cancer.

**Conclusion:**

The trends found in this USA study suggest prostate cancer may be partially correlated with meteorological factors. The patterns observed were consistent with what we would expect given the effects of climate on the deposition, absorption, and degradation of persistent organic pollutants including pesticides. Some of these pollutants are known endocrine disruptors and have been associated with prostate cancer.

## Background

**Figure 1:**
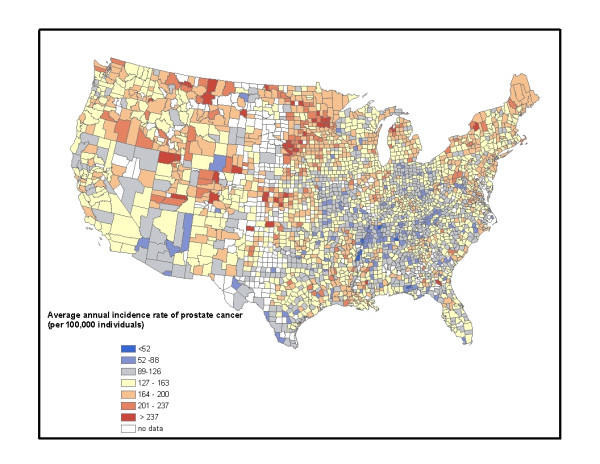
**Average annual age-adjusted incidence rate of prostate cancer for Caucasians in the U.S. between 2000-2004**. The counties with no color either have no data or counts less than 5. Data were obtained from the National Cancer Institute.

Approximately one in six men will develop prostate cancer in their life-time [1]. However, the risk of prostate cancer is not the same across the United States; northern counties tend to have a higher incidence of the disease than southern counties [2-5] (Figure 1). This north-south pattern in prostate cancer has also been reported in other areas of the world [2]. The current hypothesis for this distribution is that lower exposure to ultraviolet (UV) radiation in the northern states, especially during the winter months, results in lower vitamin D synthesis [2,3,5-8]. This vitamin regulates transcription in cells with vitamin D receptors and therefore insufficient levels may increase the risk of prostate cancer [6,9]. A recent U.S. study on prostate cancer found approximately 5.5% of the variation in this disease could be explained by the UV index [3]; however, this study did not control for potential confounders.

Several other spatially-distributed factors may contribute to the north-south disease pattern. For example, meteorological parameters such as air temperature, snowfall, and rainfall all vary spatially and it is well documented that these parameters affect the deposition, absorption, degradation of persistent organic pollutants (POPs) [10-13]. Cold trapping and snow scavenging are believed to be the reason why some POPs are found at higher concentration with increasing northern latitudes [10,13,14], and may therefore play a significant role in the level of pollutants to which individuals in different geographical areas are exposed. The purpose of this study was to determine whether there was a correlation between meteorological parameters and county-level incidence rates of prostate cancer in the U.S., controlling for exposure to local pesticide use, air pollution, and other known risk factors for prostate cancer that may vary spatially.

## Results

The average annual incidence rate of prostate cancer in this study ranged between 39.5 and 311.1 cases per 100,000. Comparison of the mean incidence rate of prostate cancer for counties in the upper and lower quartiles for different meteorological parameters suggested there was a difference in cancer rates between these groups for individual climate parameters not controlling for other variables (Table 1).

**Table 1:** Average annual incidence rate of prostate cancer per 100,000 for counties within the first and third quartiles of pollution indices and meteorological parameters used in this study.

	First quartile	Third quartile
Shortwave radiation	164.97 (1.41)*	141.35(1.14)

HDD	134.62(1.22)	168.11(1.39)

Snowfall	135.04(1.24)	163.66(1.42)

Rainfall	164.69 (1.60)	131.82(1.32)

Permitted air emissions	142.96(1.09)	149.01(1.12)

Acres of land used to grow crops	142.09(1.29)	157.14(1.53)

*(standard error of the mean)

**Figure 2:**
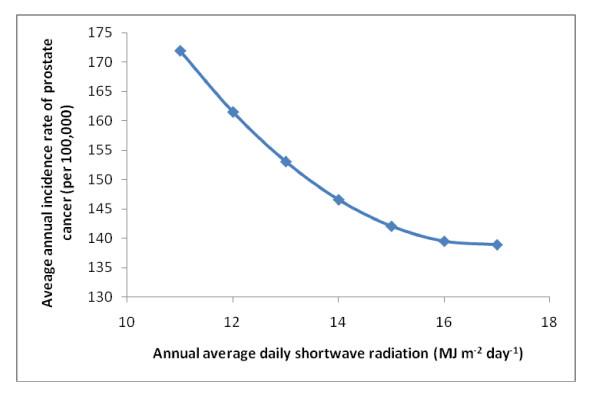
**Average annual incidence rate of prostate cancer for different levels of shortwave radiation**. Data were based on the final regression model Y = 460 - 0.198 HRT DS - 1.50 UNEMPLOY - 0.000010 CROP - 33.1 RAD - 0.00834 HDD + 0.0079 SNOW - 0.117 RAIN + 0.000002 HDD^2 ^+ 0.985 RAD^2 ^+ 0.00000046 CROP X SNOW.

**Figure 3:**
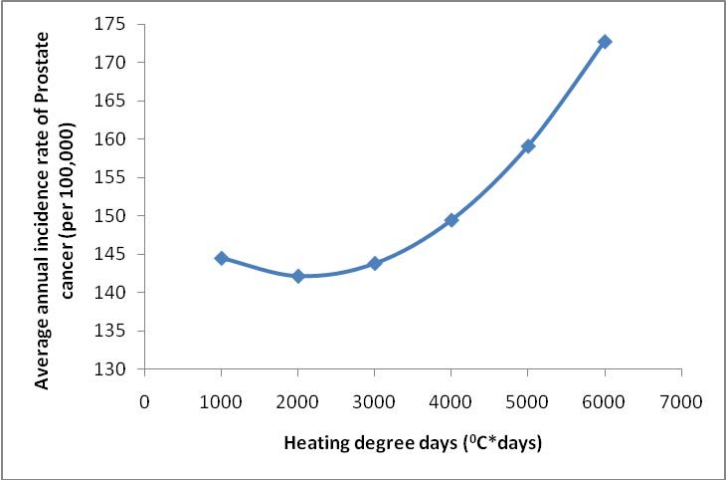
**Average annual incidence rate of prostate cancer for different levels of heating degree days (HDD)**. Data were based on the final regression model Y = 460 - 0.198 HRT DS - 1.50 UNEMPLOY - 0.000010 CROP - 33.1 RAD - 0.00834 HDD + 0.0079 SNOW - 0.117 RAIN + 0.000002 HDD^2 ^+ 0.985 RAD^2 ^+ 0.00000046 CROP X SNOW.

**Figure 4:**
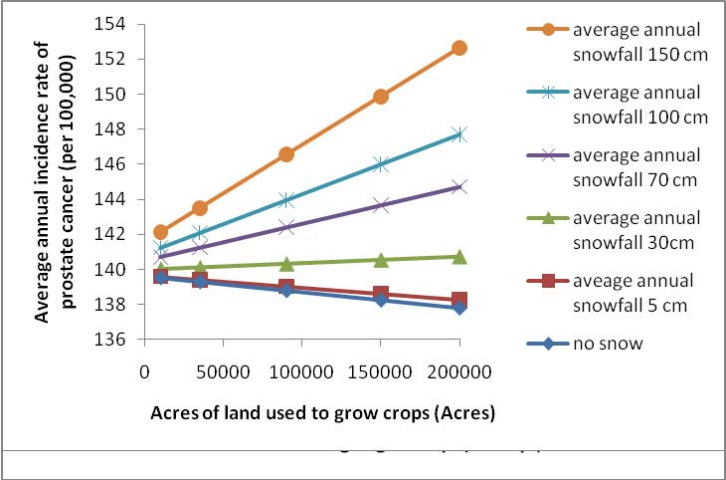
**Average annual incidence rate of prostate cancer for different levels of acres of land used to grow crops at different levels of snowfall**. Data were based on the final regression model Y = 460 - 0.198 HRT DS - 1.50 UNEMPLOY - 0.000010 CROP - 33.1 RAD - 0.00834 HDD + 0.0079 SNOW - 0.117 RAIN + 0.000002 HDD^2 ^+ 0.985 RAD^2 ^+ 0.00000046 CROP X SNOW.

Various biologically relevant models were developed using ordinary least squares (OLS) regressions (Table 2). These were developed by building on previous published models with one parameter, UV radiation. The last model we constructed, which included all significant variables available to us (Table 3) suggested radiation and temperature were best modeled using a quadratic term, and several parameters beside UV radiation were correlated with the incidence of prostate cancer (Table 3). In all our models that included meteorological parameters, UV radiation, rainfall, and temperature were always negatively correlated with prostate cancer (Table 2; Figure 2 and 3). Note that HDD is positively associated with prostate cancer, which reflects a negative correlation between temperature and the disease. The higher the HDD value the colder the county. Our index for pesticide use (acres of land used to grow crops) was positively correlated with prostate cancer, but only in counties where there was snow (Figure 4). The potential confounders in our model included premature death from heart disease and unemployment rate. These were both negatively correlated with prostate cancer in all our models (Table 2). Variables that were not significant in our models included EPA permitted air emissions for various pollutants, number of individuals residing in each county, and all interaction terms evaluated between meteorological parameters and pollution indices except acres used to grow crops crossed with snow.

**Table 2:** Equations for biologically relevant candidate models containing only significant variables in ordinary least squares regression and the corresponding AIC_C _and R^2 ^when these models were fitted using a Geographically Weighted Regression model.

Model Description	OLS regression equation for model	GWR AIC_C_ [AIC_model_- AIC_best model_]*	GWR R^2^(adj R^2^)
radiation only	Y = 240 - 6.50 RAD	18943.51 [172.91]	30.9% (29.9)

radiation with quadratic term	Y = 1101 - 122 RAD + 3.83 RAD^2^	18913.39 [142.79]	32.5%(31.1)

Pollutant and confounders	Y = 179 - .364 HRT_DS -1.477 UNEMPLOY + .00003CROP	18905.71 [135.11]	34.9%(32.4)

Radiation, confounders, and pollutant	Prst = 823.62 - .275 HRT_DS -2 UNEMPLOY -2.6RAD+.00003CROP+2.6 RAD^2^	18876.93 [106.33]	37.1% (34.0)

radiation and confounders	Y = 840.54 - .293HRT_DS -2.36 UNEMPLOY - 83.54RAD + 2.63 RAD^2^	18868.70 [98.10]	36.0% (33.4)

HDD and confounders	Y = 170 - 0.234 HRT_DS - 1.84 UNEMPLOY - 0.00550 HDD + 0.000002 HDD^2^	18843.07 [72.47]	36.4%(33.9)

Meteorological parameters without radiation but with confounders	Y = 178.3 - .21 HRT_DS -1.77 UNEMPLOY - .006HDD + .027SNOW -0.085RAIN + 0.000002 HDD^2^	18812.07 [41.47]	39.0% (35.8)

Meteorological parameters including radiation and confounders	Y = 467 - .193 HRT_DS - 1.83 UNEMPLOY -31.7RAD - .0094HDD - .16RAIN+.000002 HDD^2^ +.9 RAD^2^	18777.54 [6.94]	40.3% (36.8)

Meteorological parameters including radiation and pollutant and confounders	Y = 470 - .193 HRT_DS -1.78 UNEMPLOY -34RAD - .009HDD + .00002CROP +.03SNOW - .116RAIN + .000002 HDD^2 ^+ 1 RAD^2^	18776.77 [6.17]	42.5%(38.1)

Meteorological parameters including radiation and pollutant and confounders and interactions	Y = 460 - 0.198 HRT DS - 1.50 UNEMPLOY - 0.000010 CROP - 33.1 RAD - 0.00834 HDD + 0.0079 SNOW - 0.117 RAIN + 0.000002 HDD^2 ^+ 0.985 RAD^2 ^+ 0.00000046 CROP X SNOW	18770.60	43.3% (38.7)

* [Difference between the AIC of the model and the AIC of the best fit model. A number greater than 6 is considered significant [44]].

**Figure 5:**
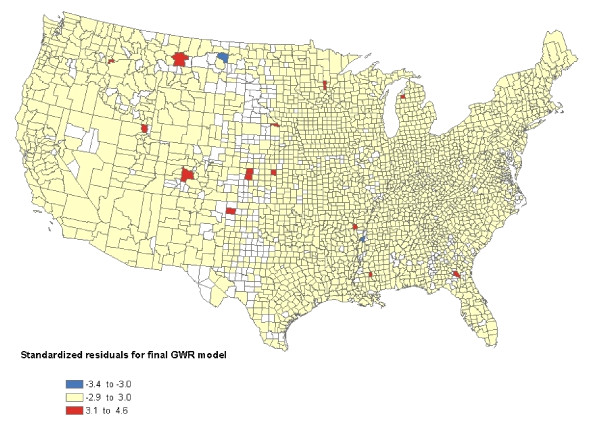
**The standardized residuals for our best fit geographic weighted regression model**. The model is described by the following equation Y = 460 - 0.198 HRT DS - 1.50 UNEMPLOY - 0.000010 CROP - 33.1 RAD - 0.00834 HDD + 0.0079 SNOW - 0.117 RAIN + 0.000002 HDD^2 ^+ 0.985 RAD^2 ^+ 0.00000046 CROP X SNOW. There was less than 0.58% of the counties with standardized residuals greater than 3 standard deviations above (indicated dark red) or below (indicated blue) the mean for the best fit GWR model.

Of the models developed using OLS analyses, the best fit model in a geographically weighted regression (GWR) analysis, based on the Akaike's Information criteria (AIC), included meteorological parameters (shortwave radiation, radiation^2^, HDD, HDD^2^, snowfall, and rainfall), confounders (premature mortality associated with heart disease and unemployment rate), the pollution index for pesticide use (acres of land used to grow crops), and the interaction term acres of land used to grow crops crossed with annual snowfall (last model in Table 2). This model explained approximately 43% of the variation in the county level incidence rate of prostate cancer (R^2 ^= 0.43) (Table 2). In comparison, the GWR model with only shortwave radiation explained approximately 31% of the variation in prostate cancer (R^2 ^= 0.31) (Table 2). The modeling assumptions for the final model with the lowest AIC were satisfied, the residuals were approximately normal and there were fewer than 0.58% (16/2571) of the counties with standardized residuals greater than 3 standard deviations above or below the mean (Figure 5). Further, the counties with these extreme residuals were scattered throughout the U.S. and did not cluster in a particular area.

## Discussion

Our analyses suggest meteorological conditions, including daily shortwave radiation, heating degree days (HDD), which is defined as the annual sum of degrees Celsius required to attain 18.3 °C when the air temperature is less than 18.3°C, and average annual snowfall and rainfall, were significantly correlated with the average annual county-level incidence rates of prostate cancer (Tables 2 and 3). This study confirmed the negative correlation between shortwave radiation and the incidence of prostate cancer (Tables 2 and 3). This was consistent with previous analyses and with the hypothesis that lower exposure to UV radiation results in lower Vitamin D synthesis [2,3,5-8]. UV radiation may also reduce the risk of cancer by increasing the photodegradation of some chemicals, including pesticides [15,16]. We improved the previously described UV model for prostate cancer [3] by including a quadratic term, which suggests there may be an upper threshold effect to the benefit of UV radiation (Figure 2). However, even with this parameter in our model, other meteorological variables appear to be significantly correlated with this cancer.

**Table 3:** β-Coefficients for final ordinary least squares regression model including meteorological parameters, confounders, pollution indices, and the significant interaction term*.

Predictor	Coefficient	P
Constant	460.30	<0.001

Heart disease mortality	-0.19781	<0.001

Average annual unemployment rate	- 1.5025	<0.001

Acres of land used to grow crops	-0.00001040	0.188

Shortwave radiation	- 33.10	0.011

Heating degree days	- 0.008341	<0.001

Average annual snowfall	0.00789	0.636

Average annual rainfall	-0.11708	<0.001

Heating degree days squared	0.00000157	<0.001

Shortwave radiation squared	0.9851	0.021

Interaction term (Crop)(Snow)	0.00000046	<0.001

*This model explained approximately 18.5% of the variation in the county level average annual incidence rate of prostate cancer (R^2 ^= 0.185) and had the lowest AIC in the GWR analysis.

Temperature was negatively correlated with prostate cancer, after controlling for shortwave radiation, local pesticide use, rainfall, snowfall, premature mortality from heart disease, and unemployment rate. At 3000 degree days, the median value for HDD in our dataset, or higher our final OLS regression model suggests a positive relationship between HDD and the incidence of prostate cancer: the higher the HDD the colder the county (Table 3 and Figure 3). A quadratic term best described the relationship between HDD and prostate cancer (Table 3). Based on our OLS model the correlation between temperature and prostate cancer was biologically negligible for counties with less than 3000 HDD, but after the HDD reached this threshold it was positively correlated with prostate cancer (Figure 3). Interestingly, the model with only HDD and the confounders (premature mortality from heart disease and unemployment) had a lower AIC than the model with only shortwave radiation and the same confounders (Table 2).

We hypothesize that temperature may be associated with the incidence of prostate cancer by modulating exposure to POPs, some of which have been linked to the disease. Temperature affects POPs in a number of ways. For example, cold temperature increases the solid phase portioning of POPs [10]. Organic chemicals, especially semi-volatile organic contaminants such as polychlorinated biphenyls (PCBs), polycyclic aromatic hydrocarbons (PAHs), and organophosphate and organochlorines pesticides, favor a solid phase rather than a gaseous phase at cold temperatures, which causes them to precipitate to the earth's surface [10,13,14]. Cold trapping of chemicals partially explains the presence of PCBs and other pollutants in pristine areas at high altitude and latitude [10,12,17]. Some semi-volatile compounds are known to be endocrine disruptors (i.e. PCBs, Alpha HCH, gamma HCH, PeCB HCB, and alpha endosulphans) [18,19], and their increased deposition at colder temperatures may predispose these places to endocrine responsive diseases (i.e. prostate cancer). Similar volatilization occurs with some persistent organic pesticides [20]. Several pesticides have been identified as endocrine disruptors [21] and have been associated with prostate cancer [22-26].

Temperature also affects the degradation of POPs in the soil and the atmosphere [27,28]. Experiments have demonstrated that the biodegradation of certain organic compounds by microorganisms is temperature-dependant and slower at colder temperatures [26]. Chemical reactions in general are slower at colder temperatures. Lower degradation of POPs at northerly latitudes suggests that environmental bioaccumulation of some pollutants may be greater in the northern part of the U.S. than in the south where temperature-dependant biodegradation processes are more productive.

Humidity also plays an important role in absorption and degradation of POPs. In general, the higher the humidity the greater the absorption and degradation of non-polar semi-volatile compounds, such as PCBs, PAHs, and the less volatilization of these compounds [27-31]. We observed a strong negative correlation between the incidence of prostate cancer and rainfall (Tables 1 and 2), which may reflect the increase in absorption and degradation of organic pollutants in moist soils and the decrease in volatilization of these compounds in humid environments [21,28].

In all our models the amount of land used to grow crops was significantly correlated with prostate cancer (Table 2). This is consistent with several studies that have found some types of pesticides associated with this cancer [22-25]. Interestingly this relationship was stronger in the counties with a high average annual snowfall (> 40 cm/year). Areas with low average annual snowfall did not have a significant relationship between land used to grow crops and prostate cancer (Figure 4).

There may be several possible explanations for the interaction between acres of land used to grow crops and average annual snowfall. Snowflakes have a high surface area-to-volume ratio and, as they fall through the atmosphere, they scavenge and collect small particulate matter, including PCBs and PAHs (suspected endocrine disruptors) [13]. Snow trapping of atmospheric pollutants may compound the effect of pesticides by increasing the deposition of POPs. Also associated with snow are cold temperatures, which reduced biodegradation of chemicals [27,28].

It is also likely that areas with different climates grow different crops that require different pesticides. Refining the crop variable to include different types of crops may help clarify this relationship. Obtaining specific measures on type and quantity of pesticides used in each county would also clarify this association. Currently this information is not collected for all counties in the U.S.

We initially controlled for the effect of the local permitted air emissions and the number of individuals living in each county; however, these variables were never significantly correlated with the incidence of prostate cancer (p values were always greater than 0.185 for all OLS models). It is possible that our measure of air pollution, which was an aggregation of over 350 permitted chemicals, was too crude. Refining the air emissions variable to test individual chemicals or groups of compounds that have similar mechanisms of action and chemical properties may identify specific air contaminants that are problematic for prostate cancer.

Another limitation of this study was the fact that we could not include many known risk factors for prostate cancer because the data were not available at a county level for all of the U.S. Of particular concern to us was the omission of ethnicity and obesity because both of these variables are known to cluster spatially and are associated with prostate cancer [32,33] thus they had the potential to distort the correlations between the meteorological parameters and the incidence of prostate cancer. Despite not being able to control for these potential confounders directly other parameters such as race, premature mortality from heart disease, and unemployment rates were controlled. Controlling for these other confounders may have inadvertently controlled for the effects of ethnicity and obesity.

Although premature mortality from heart disease and unemployment rates were only included in this study to control for confounding bias, their correlation with prostate cancer is noteworthy and suggests these variables should be included in future models, as both were negatively correlated with the incidence of prostate cancer. We found the more premature heart disease in a county the less prostate cancer there was and, likewise, the more unemployment (lower socioeconomic status) the less prostate cancer in a given county.

Besides controlling for socioeconomic status by including unemployment rate in our model we also tried to minimize the effect of this variable on our outcome parameter (prostate cancer) by using incidence rates instead of mortality rates. We believe mortality rates are influenced by the treatment an individual receives, and this is influenced by their socioeconomic status. The diagnosis of prostate cancer is initially dependant on screening, which is also associated with the individual's socioeconomic status, but presumably an individual with advanced stages of prostate cancer will be diagnosed regardless of their socioeconomic status.

Because all variables were measured and analyzed at the county level in this study, there was potential for bias if individuals in the counties were not actually exposed to the factors included in the model. Given the long incubation period of prostate cancer it is possible that some individuals with this disease migrated between counties during this period, which would not be reflected in our measure of exposure to meteorological parameters. This may have been problematic in our study because older individuals, who are at higher risk of prostate cancer [34], are more likely to emigrate in one direction: north to south. If anything, this misclassification of individuals would have biased our findings towards the null. It should be noted that to properly control for this type of bias would require conducting studies using data that are collected at the individual level.

Despite the limitations of this study, for example, the fact that some variables were only crude measures of the parameters of interest, that we may have left out some confounding variables, and that all variables were aggregated at the county level, it provides preliminary data suggesting there are correlations between meteorological parameters and prostate cancer. Regardless of the biological parameters included in our models, temperature, shortwave radiation and rainfall were always significant (Table 2). Although it is not possible to determine why meteorological conditions are correlated with prostate cancer using an ecological study, the trends detected in this study are consistent with the literature on environmental chemistry, which suggests meteorological parameters may predispose northern climates to higher levels of pollutants. The transportation and deposition of global sources of POPs to areas with colder temperatures, the reduced efficiency of degradation of compounds in cold dry climates, and the increased volatilization of POPs at low humidity may expose northern regions to higher levels of pollutants. This study, therefore, provides an additional hypothesis for the north-south distribution of prostate cancer, which builds on the existing supposition that individuals at northern latitudes are deficient in Vitamin D because of the low exposure to UV radiation during the winter months. Our study suggests that other meteorological conditions may also significantly affect the incidence of prostate cancer in a county. The findings from this study warrant further investigation using a study design that can more definitively measure the associations between meteorological parameters, and their effects on pollution and prostate cancer.

## Methods

### Methods and Results

#### Data Collection

We extracted Caucasian average age-adjusted annual incidence rates (cases per 100,000 population per year) of prostate cancer between 2000 and 2004 from the National Cancer Institute (NCI) [35], for each county in the United States. Analyses were only performed on data for Caucasians (of Hispanic and non-Hispanic origin combined) to control for the effect of race. All data were adjusted to the 2000 U.S. standard population. For six states, including Illinois, Maryland, Minnesota, Mississippi, Tennessee, and Virginia, we obtained data from individual State Cancer Registry websites, as their data were not available through the NCI. For the state of Illinois where data were only available for all races combined, only data from counties where more than 95% of the population was Caucasian were included. We assumed the rates were representative of Caucasians in these cases. We excluded counties with average annual prostate cancer counts of less than 5 from the analysis because stable accurate age-adjusted rates were not available for these counties. The time block used to calculate the average annual age-adjusted incidence rate varied slightly by states (i.e. 2000-2004 or 2001-2005, and in one case 1999-2003); however, it was always an average for a 5 year period. Data indicated a north-south spatial distribution (Figure 1).

Given the increasing frequency of studies reporting associations between different types of pesticides and prostate cancer [22,22,24,25], we felt it was necessary to control for this variable in our models. We used acres of land used to grow crops as a proxy for pesticide use; these data were available through the U.S. Census Bureau. We also acquired population demographics from the Census Bureau [36] for the 3109 counties in the continental U.S. County level data included total population in 2000, household income for Caucasians in 1999, and annual average unemployment rate between 2000 and 2004. The annual average age-adjusted mortality rate for male Caucasians between 1 and 65 years of age in 2000 and 2004 was acquired through the Centers for Disease Control and Prevention [37].

Environmental information on average shortwave radiation, average temperature, mean heating degree days (HDD), mean cooling degree days (CDD), average number of frost days, and mean precipitation between 1980 to1997 was obtained from Daymet U.S. Data Center [38]. The spatial reference for these data was defined in ArcGIS (v. 9.3.1) using a projection file provided by the Utah State University Spatial Data Group [39]. Data were then re-projected to allow for the calculation of means by county using zonal statistics.

Average monthly snowfall data from U.S. weather stations for 2000, 2001, 2002, and 2003 were obtained from the National Climatic Data Center [40]. Stations with missing data between the months of October and May were excluded from the analysis. The average snowfall for each year was calculated for all remaining weather stations and subsequently an average was calculated for the 4 years of data. Once this summary statistic was available the weather stations were georeferenced, and using zonal statistics the average snowfall was calculated for the period between 2000 and 2003 for each county with weather station information. We then interpolated the data and estimated snowfall values for the 442 counties with missing data. These counties were randomly distributed in the Eastern and Southeastern United States. Average annual rainfall was calculated by subtracting one tenth of the average annual snowfall (converted from inches/10 to cm) from the mean 18 year average annual precipitation.

Permitted air emissions data for 2002 was obtained from the U.S. Environmental Protection Agency [41]. Emissions were reported for over 350 chemicals. We aggregated the chemicals and determined the sum of permitted emissions for each county using zonal statistics in ArcGIS (v 9.3.1).

#### Statistical analyses

Prior to creating models for prostate cancer we screened the variables for correlation because several of the variables of interest measured similar parameters. For example, average temperature, HDD, CDD and mean frost days were well correlated (Pearson r was always greater than 0.89). Given we were most interested in the effect of cold temperature on prostate cancer we chose to include HDD, which is defined as the annual sum of degrees Celsius required to attain 18.3 °C when the air temperature is less than 18.3°C.

Snowfall was positively correlated with HDD (Pearson r = 0.730) and negatively correlated with rainfall (Pearson r = -0.435), and rainfall was negatively correlated with HDD (Pearson r =-0.572). Despite the correlation between these variables we retained all of them for initial testing in our models because they measured different biological parameters.

Once we selected the potential variables to be included in our OLS regression analyses we created several biologically relevant candidate models for explaining the incidence of prostate cancer. These models included various levels of complexity. The first model was similar to what has been published by Schwartz and Hanchette [3] and included only shortwave radiation. This was used as a comparison for other models. We subsequently added potential confounders such as premature mortality from heart disease and county unemployment rate, as well as higher order terms for shortwave radiation and HDD to account for curvature (Table 2). We also tested a model that included all meteorological parameters, potential confounders, and pollution indices (air emissions, acres of land used for crops, and population). The most extensive model tested included all meteorological variables, pollution indices, confounders, and biologically relevant interaction terms between meteorological parameters and pollution indices. Only variables with p values less than 0.05 were considered significant and maintained in any models.

Subsequently, all candidate models were fitted using GWR analyses [42]. These analyses use information from surrounding counties to build a model where the relationship between the dependent variable and independent variables varies spatially. An adaptive kernel type function using 10% of the U.S. counties as our distance criterion was used for all GWR analyzes. This large distance criteria was required because of the co-linearity between the numerous variables in our model, however the influence of the variables on the outcome was weighted by distance [42]. These models were conducted in Spatial Analysis in Macroecology (v 3.0) [43]. The best fit model in our GWR analyses was determined using the AIC [44]. The residuals from this model were standardized (subtracted from the mean and divided by the number of observations) and mapped in ArcGIS (v 9.3.1).

To clarify the relationship between prostate cancer and the statistically significant interaction term as well as the quadratic terms in the best fit GWR model we used the OLS regression equation from our best fit model and graphed the relationships. These plots were generated by introducing the median value for all parameters except those of interest and determining the incidence of prostate cancer associated with the upper and lower quartile range of values for the parameter(s) of interest (Figures 2, 3, and 4). The figures were generated in Excel (2007 Microsoft^® ^Office Excel^® ^2007).

## Competing interests

The authors declare that they have no competing interests.

## Authors' contributions

SS provided the idea for the project, assisted with the data interpretation, and helped write the manuscript. RM extracted the cancer and health data. SM and AC extracted and geo-referenced all the environmental data. DD conducted all the statistical analyses and helped with the interpretation of the data. All authors participated in the review and final approval of the manuscript.
